# Neurophysiology of the “Celiac Brain”: Disentangling Gut-Brain Connections

**DOI:** 10.3389/fnins.2017.00498

**Published:** 2017-09-05

**Authors:** Manuela Pennisi, Alessia Bramanti, Mariagiovanna Cantone, Giovanni Pennisi, Rita Bella, Giuseppe Lanza

**Affiliations:** ^1^Spinal Unit, Ospedale Cannizzaro Catania, Italy; ^2^Centro Neurolesi Bonino Pulejo (IRCCS) Messina, Italy; ^3^Department of Neurology IC, Oasi Maria SS (IRCCS) Troina, Italy; ^4^Department of Surgery and Medical-Surgical Specialties, University of Catania Catania, Italy; ^5^Section of Neurosciences, Department of Medical and Surgical Sciences and Advanced Technology, University of Catania Catania, Italy

**Keywords:** celiac disease, cortical excitability, electroencephalography, evoked potentials, transcranial magnetic stimulation, neuroplasticity

## Abstract

Celiac disease (CD) can be considered a complex multi-organ disorder with highly variable extra-intestinal, including neurological, involvement. Cerebellar ataxia, peripheral neuropathy, seizures, headache, cognitive impairment, and neuropsychiatric diseases are complications frequently reported. These manifestations may be present at the onset of the typical disease or become clinically evident during its course. However, CD subjects with subclinical neurological involvement have also been described, as well as patients with clear central and/or peripheral nervous system and intestinal histopathological disease features in the absence of typical CD manifestations. Based on these considerations, a sensitive and specific diagnostic method that is able to detect early disease process, progression, and complications is desirable. In this context, neurophysiological techniques play a crucial role in the non-invasive assessment of central nervous system (CNS) excitability and conductivity. Moreover, some of these tools are known for their valuable role in early diagnosis and follow-up of several neurological diseases or systemic disorders, such as CD with nervous system involvement, even at the subclinical level. This review provides an up-to-date summary of the neurophysiological basis of CD using electroencephalography (EEG), multimodal evoked potentials, and transcranial magnetic stimulation (TMS). The evidence examined here seems to converge on an overall profile of “hyperexcitable celiac brain,” which partially recovers after institution of a gluten-free diet (GFD). The main translational correlate is that in case of subclinical neurological involvement or overt unexplained symptoms, neurophysiology could contribute to the diagnosis, assessment, and monitoring of a potentially underlying CD.

## Introduction

Celiac disease (CD) is an autoimmune disorder triggered by the ingestion of gluten that, in genetically predisposed individuals, leads to damage of the small intestine and consequent malabsorption. Most patients (95% of them) are carriers of the DQ2 or DQ8 haplotype of the histocompatibility complex class II human leukocyte antigen (Lebwohl et al., 2017). Tissue transglutaminase (tTG) is the main auto-antigen (Alaedini and Green, 2008), whereas gut histopathology shows variable degrees of small bowel mucosal villi atrophy. CD affects 0.3–1.5% of the general population (approximately 1 of 120–300 people in Europe and America) (Morello et al., 2003; Bingley et al., 2004). To date, the only established therapy is a lifetime dietary gluten restriction, which is usually followed by relief of several clinical manifestations, normalization of histological and serological markers, as well as decreased risk of associated malignant and non-malignant complications (Holmes, 1996, 2002).

Clinically, although diarrhea and other gastro-intestinal symptoms can commonly be observed at disease onset or in its early phases in both pediatric patients and young adults, they are not as frequent as in the past (Campagna et al., 2017). Many adults (>50%) exhibit significant extra-intestinal involvement even without typical CD manifestations (Cooke and Smith, 1966; Hadjivassiliou et al., 2002a, 2010, 2014; Bushara, 2005; Uygur-Bayramicli and Ozel, 2011; Castillo et al., 2015). Therefore, CD is considered a complex systemic disorder with multifactorial pathogenesis that should be investigated from genetic, biological, and environmental perspectives.

Currently, little is known about the neurophysiology of central nervous system (CNS) damage in CD. This review aims to summarize the electrophysiological evidence on CNS functioning and pathology, including the clinical and instrumental response to a gluten-free diet (GFD).

## Neurological complications of celiac disease: a brief overview

Nowadays, it is widely accepted that the typical disease represents a small proportion of the so-called “CD iceberg,” because 5–6 fold more patients present with atypical or silent forms (Green et al., 2005). Neurological manifestations may either precede or follow the disease, or be present at its onset (Hadjivassiliou et al., 2002a, 2010, 2014; Briani et al., 2008). Therefore, a sensitive and specific diagnostic method able to recognize early disease process, progression, and complication would be desirable.

To date, both the causative factors and pathophysiological mechanisms of neurological involvement in CD remain a matter of debate. According to the literature, the nervous system may be one of the elective sites of gluten-mediated pathogenesis, including cross-reacting antibodies, immune-complex deposition, direct neurotoxicity, other immune-mediated factors, and deficiency of vitamin and other nutrients secondary to chronic malabsorption (Zelnik et al., 2004; Bushara, 2005; Abenavoli, 2010; Parisi et al., 2015). Recently, studies using single photon emission computed tomography showed regional changes in cerebral perfusion, with regression after institution of a GFD (De Santis et al., 1997; Usai et al., 2004). The authors argued that the cerebral hypoperfusion might be related to intestinal hyperemia from immune-mediated or endothelial damage due to immune-complex deposition likely involving antibodies against gliadin (De Santis et al., 1997). Alternatively, cortical brain hypoperfusion could reflect focal vasculitis secondary to perivascular inflammation (Usai et al., 2004).

### Cerebellar ataxia

“Gluten ataxia” is one of the first recognized symptoms (Cooke and Smith, 1966) and the most frequent neurological disturbance in CD (Hadjivassiliou et al., 2015). Dysarthria, cortical-spinal signs, eye and gaze movement disorders, and cerebellar ataxia are representative presentations. Recent studies showed deposits of antibodies against tTG on cerebellar blood vessels, adding support to a blood-brain barrier (BBB) dysfunction in CD (Hadjivassiliou et al., 2006, 2015). Interestingly, gluten ataxia is not usually related to intestinal manifestations or vitamin deficiency, and improvement with a GFD is possible (Hadjivassiliou et al., 2006).

### Peripheral neuropathy

Peripheral neuropathy is the second most common neurological manifestation of CD (up to half of patients) after cerebellar ataxia, and can appear even before diagnosis (Chin et al., 2003; Chin and Latov, 2005). Studies on the effect of a GFD on peripheral neuropathy are conflicting, with some authors reporting clinical improvement whereas others concluding a lack of relevant response (Luostarinen et al., 2003; Siqueira Neto et al., 2004). A previous study on 32 consecutive adult patients complaining of peripheral neuropathy, autonomic dysfunction, or both, and showing anti-neuronal antibodies found no response despite the adoption of a GFD (Tursi et al., 2006).

### Epilepsy

A bidirectional link between epilepsy and CD has been established in several studies, although not all (Vieira et al., 2013), with rates of prevalence from 3.5 to 7.2% (Cooke and Smith, 1966; Zelnik et al., 2004; Bushara, 2005; Uygur-Bayramicli and Ozel, 2011; Hadjivassiliou et al., 2014; Parisi et al., 2015). A large population-based cohort study observed an increased risk of CD in subjects of all ages, including children, even when epilepsy was independently restricted to patients receiving the diagnosis of epilepsy and those with prescriptions of antiepileptic drugs (Ludvigsson et al., 2012). The hypotheses accounting for epilepsy in CD included a gluten-mediated toxicity, an immune-induced cortical damage, the presence of cerebral calcifications, and vitamins/trace elements malabsorption. GFD usually controls seizures refractory to antiepileptic drugs (Hadjivassiliou et al., 2002a; Canales et al., 2006). CD-related progressive ataxia is associated with stimulus-sensitive myoclonus, opsoclonus-myoclonus, and sometimes with seizures (Borg, 2006; Deconinck et al., 2006).

### Headache

It has been reported that a GFD results in complaints of less severe headache symptoms by celiac patients (Hadjivassiliou et al., 2001). Accordingly, structural and functional neuroimaging studies were in favor of an association between migraine and CD, with relief after gluten restriction (Hadjivassiliou et al., 2001). However, when headaches in CD patients were compared with those in the general population, a conclusive association was not proven (Nikpour, 2012).

### Cognitive impairment and dementia

Adult CD patients often complain of mild cognitive symptoms called “brain fog,” which improves when gluten-restriction is started, but re-appears with dietary contamination (Lichtwark et al., 2014; Yelland, 2017). Concentration and attention difficulties, episodic memory deficits, word-retrieval problems, reduced mental acuity, and episodes of confusion or disorientation are the commonly reported features (Lurie et al., 2008). In some severely affected patients, dementia can develop as acalculia, confusion, amnesia, and personality disorders (Collin et al., 1991; Hu et al., 2006; Lurie et al., 2008; Casella et al., 2012). Despite long-term administration of a GFD, patients older than 65 years exhibited worse cognitive performance than age- and sex-matched controls (Casella et al., 2012).

### Neuropsychiatric disorders

Several psychiatric symptoms, including depression, bipolar disorder, apathy (Carta et al., 2003, 2015; Cicarelli et al., 2003), excessive anxiety (Bushara, 2005; Campagna et al., 2017), irritability (Hernanz and Polanco, 1991), schizophrenia (De Santis et al., 1997; Bushara, 2005), eating disorders (Addolorato et al., 2001), attention-deficit/hyperactivity disorder (Karwautz et al., 2008), autism (Niederhofer and Pittschieler, 2006), and sleep complaints (Barcia et al., 2008) have been associated with CD.

Reactive anxiety that usually ameliorates with a GFD is the predominant form of anxiety disorder in these patients. Depressive disturbances, which affect a relevant number of subjects, may significantly impair quality of life, and are a good predictor of lack of dietary compliance (Zingone et al., 2010). Therefore, screening patients for depression is of pivotal importance both at diagnosis and follow-up in order to advice psychological support and/or pharmacological therapy. Possible causative factors of mood disorders might be tryptophan deficiency secondary to chronic malabsorption (Hallert et al., 1982; Hernanz and Polanco, 1991) or co-morbidity with thyroid disease (Carta et al., 2002). Decreases in levels of serotonin, dopamine, and noradrenaline metabolites in cerebrospinal fluid as well as tryptophan and other monoamine precursors in serum were observed in untreated patients (Hallert et al., 1982; Hernanz and Polanco, 1991).

Clinical improvement was reported only after long-term administration of a GFD (>5 years) (van Hees et al., 2013), highlighting the importance of prolonged alimentary restriction on extra-intestinal CD symptoms as well.

## Neurophysiological techniques used to probe CNS involvement in celiac disease

### Electroencephalography

The spectrum of electroencephalography (EEG) features associated with CD is rather wide, although focal activity in terms of unilateral or bilateral spike or slow waves, mainly localized in the occipital regions, have been reported in most of the wakefulness EEG studies (Magaudda et al., 1993; Labate et al., 2001; Pratesi et al., 2003; Ranua et al., 2005; Lionetti et al., 2010; Licchetta et al., 2011; Aksoy et al., 2016). However, as recommended (Parisi et al., 2014), EEG patterns should not be considered disease-specific.

A recent prospective study among 307 CD children compared with 197 age- and sex-matched controls observed that patients were more prone to epileptiform activities on EEG (spike/sharp-wave discharges, especially in the occipital lobes but also in the central-temporal sites and in diffuse distribution). However, early and strict adherence to a GFD effectively decreased these findings (Işıkay et al., 2015a). In addition, a positive correlation between tTG level and epileptiform changes during sleep and awake EEG was found (Işıkay et al., 2015a). The concept that the occipital region is frequently involved in CD seems to be supported by the evidence of occipital calcium deposition, occipital lobe semiology, and EEG findings. The preferential involvement of this lobe may lie on several factors, such as its vulnerability to some metabolic circumstances (e.g., hypoglycemia and hypoxia) and its thinner morphological structure than other cortical regions (Işıkay et al., 2015b). However, the opposite scenario (the occurrence of CD in patients with “posterior” epileptic semiology) is not always true because in a group of 90 pediatric epileptic patients with occipital EEG abnormalities, tTG antibody was positive in only two (Canales et al., 2006).

CD-associated epilepsy has also been reported in association with other neurological signs, such as ataxia, tremor, and progressive myoclonus (Javed et al., 2012; Sarrigiannis et al., 2014). In these cases, epilepsy was usually refractory, and EEG demonstrated spike and waves in the right anterior and mid-temporal lobes, as well as bilateral slow and sharp waves. Some of these spike waves were present in association with localized jerks of the upper or lower limb, although without periodic complexes (Javed et al., 2012; Sarrigiannis et al., 2014). Finally, the fixation-off sensitivity phenomenon could be observed (Casciato et al., 2015).

In regard to the impact of dietary restriction, a recent study in 19 children with biopsy-proven CD revealed abnormal EEG findings in 48% of them that were no longer evident in most of the patients after 6 months of GFD (Parisi et al., 2015). However, some asymptomatic children and adolescents still manifested hyperexcitability to EEG despite the diet (Parisi et al., 2015).

In summary, CD screening should be performed in patients with cryptogenic and/or refractory epilepsy, or in the presence of unexplained EEG findings. Gluten restriction is usually effective in ameliorating the clinical-instrumental correlates.

### Evoked potentials

Few studies, most of which used somatosensory evoked potentials (SEPs), have explored CD with evoked potentials. Di Lazzaro and co-workers reported a patient whose lower limb SEPs presented enlargement of lumbar waves and bilateral lack of cortical responses, suggesting an impaired somatosensory conduction along the spinal dorsal columns; GFD induced complete clinical-instrumental recovery (Di Lazzaro et al., 2010). Another case presented one child with prolonged central conduction time among 27 treated children (Cakir et al., 2007). In patients with cerebellar ataxia associated with subclinical CD responding to a GFD, normal SEPs were reported (Pellecchia et al., 1999). However, in a large cohort of adult ataxic patients, more than half had loss or delayed P40 cortical response, suggesting dorsal column degeneration (Bürk et al., 2001). In regard to SEPs in cortical mycolonus, a previous study in two CD-proven subjects with myoclonic ataxic syndrome showed giant and time-locked cortical responses that preceded the myoclonus (Tijssen et al., 2000). The authors speculated that, in spite of the neurophysiological evidence of cerebral cortical involvement, the hyperexcitability was mainly located in the cerebellum, and that the effects on sensory-motor cortex represented a remote influence from cerebellar dysfunction (Tijssen et al., 2000). Conversely, the cortical electrophysiological origin of the myoclonus was argued by other researchers who found their patients responded poorly to a GFD and worsened progressively (Lu et al., 1986; Tison et al., 1989; Bhatia et al., 1995).

Although CD may impact the auditory system (Bürk et al., 2001), brainstem auditory evoked potentials (BAEPs) and vestibular evoked myogenic potentials were reported to be normal (Pawlak-Osińska et al., 2007). More recently, it was found that only 1 of 25 patients had abnormalities in BAEPs in terms of moderate sensorineural hearing loss (Aksoy et al., 2016).

Complications affecting visual pathways may develop in CD and be evidenced by visual evoked potentials (VEPs) (Bürk et al., 2001; Freeman, 2008; Hadjivassiliou et al., 2010). In particular, patients can show abnormalities on VEPs without evident lesions at neuroimaging (Aksoy et al., 2016). A previous case described a slight increase of P100 wave latency bilaterally at pattern-reversal VEPs that reverted back to normal after a GFD (Pellecchia et al., 1999). Given their role in detecting even preclinical pathology in subjects with normal ophthalmological and brain imaging exams, VEPs may provide useful insights in neurologically asymptomatic CD patients (Aksoy et al., 2016).

### Transcranial magnetic stimulation

In 1999, Pellecchia and co-workers first reported motor evoked responses to transcranial magnetic stimulation (TMS) in a CD patient who exhibited reduced amplitude in the rectus femoris muscle that improved with diet; however, motor responses remained undetectable in the tibialis anterior muscle (Pellecchia et al., 1999). A year later, report of delayed motor response in the left tibialis anterior muscle and abnormal cortical inhibition was published in one of three CD patients with cortical myoclonus (Tijssen et al., 2000).

Specific TMS studies before and after GFD were published more recently. TMS is an electrophysiological tool able to non-invasively explore the excitation state of motor cortical areas and conductivity of the pyramidal tract *in vivo*. Moreover, it is capable of unveiling subclinical central motor involvement in different neurological and psychiatric diseases or systemic disorders affecting the CNS (Bella et al., 2011a,b, 2016; Pennisi et al., 2011a,b, 2015, 2017; Concerto et al., 2013; Lanza et al., 2013, 2015a,b, 2017a,b; Cantone et al., 2014, 2017), also providing prognostic (Bella et al., 2013; Pennisi et al., 2016) and therapeutic implications (Spampinato et al., 2013; Concerto et al., 2015; Bordet et al., 2017). Lastly, the so-called “pharmaco-TMS” can selectively probe the functioning of different central neurotransmission pathways, such as glutamate, gamma-aminobutyric-acid (GABA), monoamine, and acetylcholine, by testing their pharmacological agonists or antagonists (Paulus et al., 2008; Ziemann et al., 2015).

The first TMS study investigated 20 *de novo* CD patients without apparent neurological involvement and 20 age-matched controls (Pennisi et al., 2014). TMS revealed cortical motor disinhibition and hyperfacilitation, which is a profile compatible with dysfunctional GABAergic and glutamatergic transmissions, in patients. The authors hypothesized that an imbalance of excitatory and inhibitory circuits within the motor cortex might be the neurochemical correlate of the cross-interaction between antibodies against gliadin and specific neuronal antigens. An alternative explanation was the deposition of tTG-immunoglobulin leading to an abnormal ion levels across neuronal membrane. Likewise, antibodies synthesized within the CNS and directed against glutamic acid decarboxylase might disrupt the functioning of GABAergic interneurons (Pennisi et al., 2014).

The same cohort of patients underwent re-evaluation after a relatively short time of a GFD (median of 16 months) (Bella et al., 2015). Their gastrointestinal symptoms were ameliorated but, unexpectedly, the cortical excitability to TMS further increased. This finding was hypothesized to represent a plastic re-organization of the cerebral cortex triggered by gluten exposure and independent of GFD. On the other hand, diet duration or compliance might not have been enough to induce an adequate remission (Bella et al., 2015). A recent cross-sectional TMS study after a much longer GFD (mean period of 8.35 years) showed that a more prolonged period of gluten restriction was required to revert the cortical changes in adult CD patients. Nevertheless, regardless of diet, some specific excitatory features to TMS remained, probably suggesting an intracortical synaptic rearrangement, mostly involving glutamate-mediated interneurons (Pennisi et al., 2017).

## Discussion

**Figure 1:**
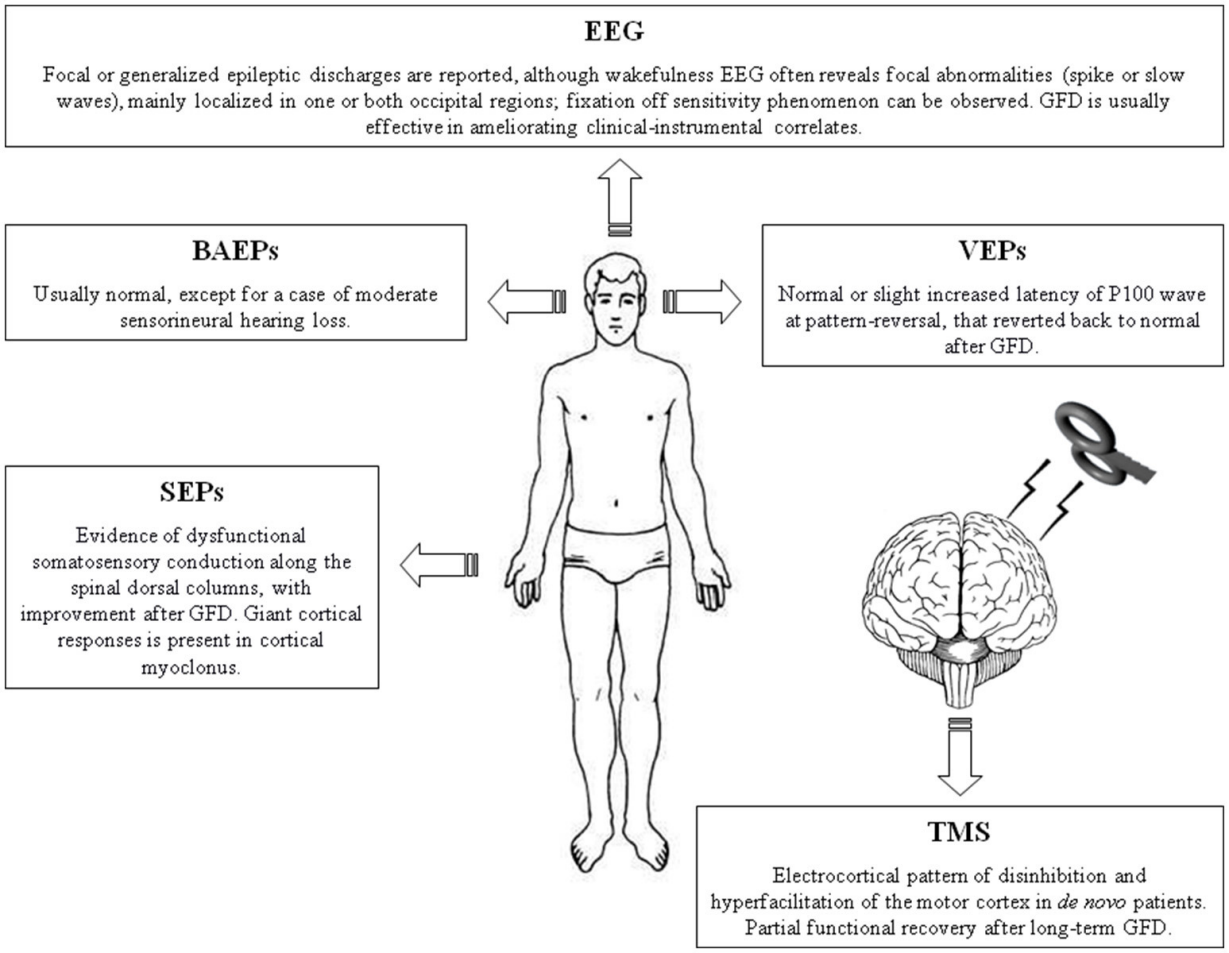
Summary figure illustrating the main neurophysiological findings in patients with celiac disease. BAEPs, brainstem auditory evoked potentials; EEG, electroencephalography. GFD, gluten-free diet; SEPs, somatosensory evoked potentials; TMS, Transcranial magnetic stimulation; VEPs, visual evoked potentials.

The main translational value of this review is that clinical neurophysiology can contribute to the diagnosis, assessment, and monitoring of CD even in patients with subclinical CNS involvement or unexplained neurological symptoms (Table 1, Figure 1). In particular, the majority of electrophysiological changes are often subclinical (“celiac iceberg”), and these need to be strictly monitored because of the possibility of progression to clinically visible neurological syndrome in both young and adult patients (“symptomatic celiac disease”). Accordingly, it has been shown that there is an increased risk of neurological complications in atypical or silent CD forms, especially in older patients or those older at diagnosis (Aksoy et al., 2016). It is worth noting that, despite their valuable role, anti-ganglioside antibodies and neuronal antigens are not always specifically linked to the neurological manifestations and their progression in the course of CD (Kaplan et al., 1988; Gobbi et al., 1992; Hadjivassiliou et al., 1998, 2002b; Aksoy et al., 2016).

**Table 1:** Studies using electrophysiological techniques probing the central nervous system involvement in patients with celiac disease.

**Study**	**Number of patients**	**Sex**	**Age (years)**	**Neurological features**	**Neurophysiology**	**Main results**	**Response to GFD**	**Translational value**
Lu et al., 1986	2	M/F	M: 42/F: 54	Action limbs myoclonus, seizures, ataxia	a) EEG b) SEPs	a) M: bilateral high-amplitude spike and polyspike discharges; F: normal b) M: cortical responses greatly enlarged that preceded spontaneous muscle jerks, consistent with cortical reflex myoclonus; F: enlarged response on contralateral sensory-motor area	No response	First evidence of electrocortical pathology in CD
Tison et al., 1989	1	F	56	Arm, neck, face stimulus-sensitive and palatal myoclonus, cerebellar ataxia	a) EEG (back averaging) b) SEPs c) VEPs d) BAEPs	a) Cortical contralateral spike slow waves preceding myoclonus b) Increased P1-N2 amplitude response c) Normal d) Normal	No response	Myoclonus as a prominent feature of CD encephalopathy
Magaudda et al., 1993	8	5 F/3 M	Mean 17.5 (range 10-23)	Epilepsy	EEG	Spike and spike-waves in one or both parietal-occipital regions	Seizures disappeared in 1 patient; 2 patients did not respond. Other data not reported	Electrophysiological finding of hyperexcitable posterior cerebral regions in CD
Bhatia et al., 1995	4	1 F/3 M	Mean 57.5 (range 44-68)	Progressive myoclonic ataxic syndrome	a) EEG (back averaging) b) SEPs	a) Cortical reflex and/or action myoclonus b) Enlarged cortical SEPs	Clinical progression despite strict diet	Although the myoclonus was cortical, the electrophysiological origin was in the cerebellum
Mumford et al., 1996	1	M	44	Myoclonic ataxia, seizures	EEG	Frequent runs of bilateral High-voltage delta wave activity	No response	Patients with progressive ataxia and myoclonus should have a biopsy for CD
Pellecchia et al., 1999	1	M	34	Progressive cerebellar ataxia	a) TMS b) VEPs c) SEPs	a) Reduced amplitude of motor responses b) Bilateral increase of P100 latency c) Normal	VEPs returned to normal after 2 years diet; partial response of TMS	Impairment of central visual and motor pathways in CD
Tijssen et al., 2000	2	M	50	Myoclonic ataxic syndrome	a) SEPs b) EEG (back averaging) c) TMS (1 patient)	a) Enlarged cortical SEPs b) Time-locked cortical potential preceding the action myoclonus c) Delayed response in the left tibialis anterior and abnormal cortical inhibition	Not reported	The enhanced excitability of sensory-motor cortex may arise as a remote effect of cerebellar pathology in CD
Fung et al., 2000	1	F	48	Unilateral limb tremor, dystonia, myoclonus, and ataxia	a) EEG b) SEPs (median nerve) e) c) Jerk-locked back averaging	a) Normal b) Giant cortical response following stimulation of the affected side c) No preceding cortical potential	Not reported	CD should be considered in patients with unexplained movement disorders and seizures
Hanagasi et al., 2001	1	F	31	Ataxia, stimulus-induced myoclonus, eye movement abnormalities	a) EEG b) BAEPs c) SEPs (tibial nerve)	a) Normal b) Normal c) Normal	Myoclonus responded well to the diet	CD as a cause of neurologic syndrome even without gastrointestinal symptoms
Bürk et al., 2001	12	7 F/5 M	Mean 55 (range 30-76)	Progressive cerebellar ataxia	a) BAEPs (10 patients) b) VEPs (10 patients) c) SEPs	a) Abnormal BAEPs in 10% b) Abnormal VEPs in 30% c) Loss or delayed P40 response in 58.3%	Not reported	Evidence of dorsal column degeneration; less frequent involvement of central visual pathway
Pratesi et al., 2003	1	M	3	Drug-resistant epilepsy	EEG	Slow background activity intermixed with frequent sharp and slow wave complexes	Progressive seizure control	Association between CD and refractory epilepsy
Cakir et al., 2007	27	18 F/9 M	Mean 11.22 ± 4.27 (SD)	Asymptomatic; isolated seizure in 3	a) EEG b) SEPs	a) Normal b) Prolonged latencies in 3.7%	Subclinical neurological changes more common in non-compliant patients	Subclinical neurological abnormalities are frequent in pediatric CD
Pawlak-Osińska et al., 2007	30	Not reported	Mean: 9.2 (range 6-18)	Gaze and optokinetic nystagmus in most of them	a) BAEPs b) VEMPs	a) Normal b) Normal	No response	Neurological signs correlated with the histopathological changes
Briani et al., 2008	71	16 M/55 F	Mean 36.7 ± 12.1 (SD)	Headache, depression, peripheral neuropathy, epilepsy (16 patients)	EEG	Not reported	No serological or electrophysiology change	No clear correlation between anti-neural reactivity and neurologic dysfunction
Sallem et al., 2009	1	F	46	Generalized seizures, myoclonus, and ataxia	EEG (sleep and wake)	Occasional generalized poly-spike wave complexes	No improvement	CD as a differential diagnosis of myoclonic ataxia and progressive cerebellar dysfunction
Di Lazzaro et al., 2010	1	M	66	Fatigability, painful cramps and mild weakness at left lower limb	SEPs (tibial nerve)	Cortical responses bilaterally absent	Clinical remission and improvement of SEPs	Evidence of dorsal column involvement. Neurological symptoms even in older CD patients
Licchetta et al., 2011	8	7 F/1 M	Mean 25.6 ± 4.85 (SD)	Progressive myoclonic epilepsy	EEG (inter-ictal) and video-EEG monitoring	Focal posterior or diffuse spike-wave discharges; poly-spike-wave complexes	5 out of 7 patients did not respond	CD as a cause of progressive myoclonic epilepsy. Peculiar involvement of the occipital lobe in CD
Javed et al., 2012	1	F	63	Late onset epilepsy, ataxia, tremor, progressive myoclonus	a) EEG b) SEPs (tibial nerve)	a) Right anterior and mid-temporal spike and waves, bilateral slow waves and sharp waves b) Giant cortical SEPs	No improvement	Refractory CD is linked to progressive neurological syndrome
Parisi et al., 2014	2 (siblings)	M/F	M: 5/F: 4	M: seizures F: iron deficiency and poor growth	Awake and sleep EEG	M: left temporal spike and wave discharges, generalized abnormal activity F: bursts of bilateral occipital spikes and diffuse polyspikes and sharp waves	No improvement	A long follow-up may be required to clarify the relationships between clinical and EEG features
Sarrigiannis et al., 2014	9	3 F/6 M	Mean 59.4 ± 10.4 (SD)	Asymmetrical irregular myoclonus at limbs and sometimes face; “Jacksonian march” (3 patients) and secondarily generalized seizure (5 patients)	a) Standard EEG b) SEPs c) Jerk-locked back averaging d) Long loop reflexes	a) PLEDs, theta and delta activity (2 patients) b) Giant SEPs (5 patients) c) Cortical myoclonus d) Altered long-loop reflexes (5 patients)	Ataxia and enteropathy improved, but myoclonus remained the most disabling feature	The clinical involvement in CD covers the whole spectrum of cortical myoclonus
Casciato et al., 2015	10	9 F/1 M	Mean 31.5 (range 18-44)	Seizures	EEG	Slow and epileptiform abnormalities over parietal-occipital and temporal regions	Decrease of seizure frequency in half of patients	“Posterior” ictal semiology, EEG patterns and drug-resistance were peculiar features in CD
Pennisi et al., 2014	20	16 F/4 M	Median 33.0 (range 24-45)	Dysthymic disorder (5 patients); anxiety (2 patients)	a) EEG b) TMS	a) Normal b) Shorter CSP, reduced ICI, enhanced ICF	GFD was not started yet (*de novo* patients)	Disinhibition and hyperfacilitation of the motor cortex. Immune system dysregulation might trigger changes of cortical excitability
Dai et al., 2014	2	1 F/1 M	M: 3 / F: 10	Tonic-clonic seizures and mild intellectual disability	EEG	Bilateral spikes and slow wave complexes in the occipital lobes, predominantly in the right hemisphere	Not reported	CD is more common among patients with occipital lobe epilepsy (often drug-resistant)
Işıkay et al., 2015a	a) 216 (newly diagnosed CD group); b) 91 (GFD group	180 F/127 M	a) Mean 10.15 ± 3.7 (SD) b) Mean 9.88 ± 4.2 (SD)	Headache in 2.9%	EEG	Epileptiform activity (spike/sharp-wave discharges) in 24 patients; among them, 21 (9.7%) were in newly diagnosed group and 3 (3.3%) in GFD group	Early strict GFD is advised in patients with epileptiform activities	CD patients are prone to epileptiform activities
Işıkay et al., 2015b	a) 43 (newly diagnosed CD group) b) 132 (formerly diagnosed group)	103 F/72 M	Mean 10.6 ± 3.8 (SD)	Headache in 31.4%	EEG	Epileptiform activity in 9.3% of newly diagnosed CD patients and in 1.5% of formerly diagnosed patients	Decrease of EEG epileptiform discharges	Increased epileptiform activity among newly-diagnosed patients; tissue transglutaminase correlated with EEG
Parisi et al., 2015	19	16 F/3 M	Mean 9.82 ± 4.09 (SD)	Headache in 36.8%; positive OSA score in 31.6%	EEG	Focal or generalized sharps and/or spikes and spike-waves in 48% of children	Headache disappeared in 72% and EEG abnormalities in 78%; negative OSA score in all	Consider atypical or silent CD in case of unexplained symptoms, sleep breathing disorder or EEG abnormalities
Bella et al., 2015	13	10 F/3 M	39 (range 24-46)	Dysthymic disorder (1 patient)	TMS	Compared to the baseline (*de novo*): - decrease of median rMT - shorter CSP - reduced ICI - enhanced ICF	Increased cortical excitability after a relatively short period of diet	Functional cortical reorganization probably compensating for disease progression
Aksoy et al., 2016	65	26 M/39 F	Mean 12.85 ± 4.23 (SD)	Intellectual disability (3 patient); ophtalmoplegia and distonia (1 patient)	a) EEG b) VEPs c) BAEPs	a) abnormal in 5 patients (focal temporal epileptic activity in 2, occipital in 1, and left hemisphere in 1; generalized in 1) b) abnormal in 7 patients (marked and bilateral in 2) c) abnormal in 1 patient (sensorineural hearing loss)	EEG improved in 3 out of 4 patients on GFD and antiepileptic drugs	Increased risk of neurological abnormalities in atypical and silent forms that involves older ages and older ages at the diagnosis
Pennisi et al., 2017	a) 20 *de novo* CD patients b) 20 CD patients on GFD	a) 4 M/16 F b) 6 M/14 F	a) Mean 35.00 ± 12.03 (SD) b) Mean 35.10 ± 6.02 (SD)	a) Dysthymic disorder (5 patients); higher score for depression, anxiety, and irritability b) Normal	TMS	- Shorter CSP in *de novo* patients than GFD patients - Smaller motor response amplitude in all patients - Reduced ICI and enhanced ICF in all patients - Increased ICF in gluten-restricted compared to non-restricted patients	A prolonged dietary regimen induced a recover of most but not all electrocortical changes	Subtle intracortical synaptic dysfunction may persist notwithstanding the GFD

*BAEPs, brainstem auditory evoked potentials; CD, Celiac disease; CSP, cortical silent period; EEG, electroencephalography; F, female; GFD, gluten-free diet; ICF, intracortical facilitation; ICI, intracortical inhibition; M, male; OSA, obstructive sleep apnea; PLEDs, Periodic Lateralized Epileptiform Discharges; rMT, resting motor threshold; SD, standard deviation; SEPs, somatosensory evoked potentials; TMS, transcranial magnetic stimulation; VEMPs, vestibular evoked myogenic potentials; VEPs, visual evoked potentials*.

From a pure neurophysiological perspective, findings from EEG, SEPs, and TMS seem to converge on an overall profile of “hyperexcitable celiac brain,” albeit this may not be confined to the cerebral cortex. Indeed, an increase in cerebral cortical excitability may arise from enhanced inputs from the cerebellum (Tijssen et al., 2000). In this context, it is important to remember that malabsorption syndrome (with the consequent deficiency of vitamins and other nutrients) probably does not account for these cortical manifestations, given that it takes place in the most severely affected patients whose intestinal mucosa are seriously damaged and do not recover after institution of a GFD (Pennisi et al., 2014).

Regarding humoral autoimmunity to neuronal antigens, deposits of anti-tTG2 and anti-tTG6 antibodies have been found not only in the small intestine but also in different CNS sites (cerebellum, pons, medulla, brain blood vessels) (Hadjivassiliou et al., 2008). Furthermore, a possible BBB lesion, secondary to diffuse infiltration of T-lymphocytes and inflammatory cells within the perivascular cuffing might expose cerebral tissues to antibodies (Hadjivassiliou et al., 2010). The result may be a vicious circle that eventually leads to a prevailing synaptic hyperexcitation and a weaker inhibition at the cortical level (Pennisi et al., 2014). The increased excitability may also be the correlate of a glutamate-induced cortical rearrangement or a dysfunctional control of GABAergic inhibitory interneurons. In particular, because glutamate is of pivotal importance in synaptic plasticity, it can be speculated that immune system dysregulation triggered by gluten ingestion, might result in a long-standing activation of post-synaptic glutamate receptors accounting for the enhanced hyperexcitability (Bella et al., 2015).

The neurophysiological-based approach to CD should take into account potential pitfalls and critical aspects related to both the techniques themselves and methodological biases in the studies reviewed here. First, as mentioned, electrophysiological changes are not disease-specific. Second, an association finding does not mean causative relationship. For instance, an association between CD and amyotrophic lateral sclerosis was previously reported in different investigations (Turner et al., 2007, 2013; Brown et al., 2010; Bersano et al., 2015; Gadoth et al., 2015) but not confirmed in a large population-based cohort study (Ludvigsson et al., 2014). Finally, it is mandatory to make a differential diagnosis between hyperexcitability-related seizures and incidental EEG findings in neurologically asymptomatic CD subjects. In the latter case, EEG changes represent a confounding factor and a long follow-up is required (Parisi et al., 2014).

The response of neurological symptoms to a GFD is still controversial. Current knowledge encompasses an initial phase when patients are “gluten-sensitive” and a subsequent stage characterized by “gluten-insensitivity” (Tursi et al., 2006). An older age at diagnosis or a prolonged period of gluten ingestion may account for persistent neurological symptoms after a relatively short period of GFD (Bella et al., 2015). Moreover, gluten restriction is not usually effective in patients with refractory CD and in those with an associated autoimmune disease or some neurological complications (Hadjivassiliou et al., 2010; Castillo et al., 2015; Campagna et al., 2017). It is reasonable to conclude that some neurological aspects improve after diet restriction whereas others persist, supporting the concept that the more prolonged the GFD, the more likely clinical and neurophysiological remission may occur. However, given that neurological impairment may develop despite an adequate adherence to a GFD (Luostarinen et al., 2003; Chin and Latov, 2005; Tursi et al., 2006; Bürk et al., 2009), other causative factors have to contribute (McKeon et al., 2014): (a) accidental minimal gluten contamination despite a good dietary compliance (Green and Jabri, 2003); (b) direct gliadin-mediated inflammatory attack; (c) other components that are independent of GFD (Tijssen et al., 2000).

Based on further understanding of the pathogenesis and treatment of CD, neurophysiology-targeted non-dietary therapies are in development (Schuppan et al., 2009). Similarly, modern rehabilitation approach involves organizational measures that promote not only clinical recovery but also a better quality of life (Sabel'nikova et al., 2013) through the support of different medical and non-medical specialists (Usanova et al., 2012).

In conclusion, neurophysiology, together with clinical, serological, and imaging data, can help in disentangling the multifaceted physiopathological and neurobiological mechanisms coupling gut and brain in CD. The eventual identification of neurophysiological markers might be useful in the diagnosis and monitoring of CD, aiming to improve the healthcare of both single subjects and the global community.

## Author contributions

All authors provided substantial contributions to the conception, drafting, critical revision for important intellectual content, final approval, and agreement to be accountable for all aspects of the work. In particular, MP and AB conceived and designed the study, MC and GL reviewed the literature and drafted the manuscript, and GP and RB critically reviewed and finalized the paper.

### Conflict of interest statement

The authors declare that the research was conducted in the absence of any commercial or financial relationships that could be construed as a potential conflict of interest.

## References

[B1] Abenavoli L. (2010). Nervous system in the gluten syndrome: a close relationship. Med. Hypotheses 74, 204–205. 10.1016/j.mehy.2009.08.012 19744798

[B2] Addolorato G. Capristo E. Ghittoni G. Valeri C. Mascianà R. Ancona C.. (2001). Anxiety but not depression decreases in coeliac patients after one-year gluten-free diet: a longitudinal study. Scand. J. Gastroenterol. 36, 502–506. 10.1080/00365520119754 11346203

[B3] Aksoy E. Tıraş-Teber S. Kansu A. Deda G. Kartal A. (2016). Neurological findings spectrum in Celiac disease. Turk. J. Pediatr. 58, 233–240. 10.24953/turkjped.2016.03.001 28266186

[B4] Alaedini A. Green P. H. (2008). Autoantibodies in celiac disease. Autoimmunity 41, 19–26. 10.1080/08916930701619219 18176861

[B5] Barcia G. Posar A. Santucci M. Parmeggiani A. (2008). Autism and coeliac disease. J. Autism Dev. Disord. 38, 407–408. 10.1007/s10803-007-0480-3 17985220

[B6] Bella R. Cantone M. Lanza G. Ferri R. Vinciguerra L. Puglisi V.. (2016). Cholinergic circuitry functioning in patients with vascular cognitive impairment–no dementia. Brain Stimul. 9, 225–233. 10.1016/j.brs.2015.09.013 26515786

[B7] Bella R. Ferri R. Cantone M. Pennisi M. Lanza G. Malaguarnera G.. (2011a). Motor cortex excitability in vascular depression. Int. J. Psychophysiol. 82, 248–253. 10.1016/j.ijpsycho.2011.09.006 21945481

[B8] Bella R. Ferri R. Lanza G. Cantone M. Pennisi M. Puglisi V.. (2013). TMS follow-up study in patients with vascular cognitive impairment-no dementia. Neurosci. Lett. 534, 155–159. 10.1016/j.neulet.2012.12.017 23274709

[B9] Bella R. Ferri R. Pennisi M. Cantone M. Lanza G. Malaguarnera G.. (2011b). Enhanced motor cortex facilitation in patients with vascular cognitive impairment-no dementia. Neurosci. Lett. 503, 171–175. 10.1016/j.neulet.2011.08.022 21875648

[B10] Bella R. Lanza G. Cantone M. Giuffrida S. Puglisi V. Vinciguerra L.. (2015). Effect of a gluten-free diet on cortical excitability in adults with Celiac disease. PLoS ONE 10:e0129218. 10.1371/journal.pone.0129218 26053324 PMC4460029

[B11] Bersano E. Stecco A. D'Alfonso S. Corrado L. Sarnelli M. F. Solara V.. (2015). Coeliac disease mimicking amyotrophic lateral sclerosis. Amyotroph. Lateral Scler. Frontotemporal Degener. 16, 277–279. 10.3109/21678421.2014.980614 25646867

[B12] Bhatia K. P. Brown P. Gregory R. Lennox G. G. Manji H. Thompson P. D.. (1995). Progressive myoclonic ataxia associated with coeliac disease. The myoclonus is of cortical origin, but the pathology is in the cerebellum. Brain 118, 1087–1093. 10.1093/brain/118.5.1087 7496772

[B13] Bingley P. J. Williams A. J. Norcross A. J. Unsworth D. J. Lock R. J. Ness A. R.. (2004). Undiagnosed coeliac disease at age seven: population based prospective birth cohort study. BMJ 328, 322–323. 10.1136/bmj.328.7435.322 14764493 PMC338097

[B14] Bordet R. Ihl R. Korczyn A. D. Lanza G. Jansa J. Hoerr R.. (2017). Towards the concept of disease-modifier in post-stroke or vascular cognitive impairment: a consensus report. BMC Med. 15:107. 10.1186/s12916-017-0869-6 28539119 PMC5444106

[B15] Borg M. (2006). Symptomatic myoclonus. Neurophysiol. Clin. 36, 309–318. 10.1016/j.neucli.2006.12.006 17336775

[B16] Briani C. Zara G. Alaedini A. Grassivaro F. Ruggero S. Toffanin E.. (2008). Neurological complications of celiac disease and autoimmune mechanisms: a prospective study. J. Neuroimmunol. 195, 171–175. 10.1016/j.jneuroim.2008.01.008 18343508

[B17] Brown K. J. Jewells V. Herfarth H. Castillo M. (2010). White matter lesions suggestive of amyotrophic lateral sclerosis attributed to celiac disease. Am. J. Neuroradiol. 31, 880–881. 10.3174/ajnr.A1826 19910450 PMC7964190

[B18] Bürk K. Bösch S. Müller C. A. Melms A. Zühlke C. Stern M.. (2001). Sporadic cerebellar ataxia associated with gluten sensitivity. Brain 124, 1013–1019. 10.1093/brain/124.5.1013 11335703

[B19] Bürk K. Farecki M. L. Lamprecht G. Roth G. Decker P. Weller M.. (2009). Neurological symptoms in patients with biopsy proven celiac disease. Mov. Disord. 24, 2358–2362. 10.1002/mds.22821 19845007

[B20] Bushara K. O. (2005). Neurologic presentation of celiac disease. Gastroenterology 128, S92–S97. 10.1053/j.gastro.2005.02.018 15825133

[B21] Cakir D. Tosun A. Polat M. Celebisoy N. Gokben S. Aydogdu S.. (2007). Subclinical neurological abnormalities in children with celiac disease receiving a gluten-free diet. J. Pediatr. Gastroenterol. Nutr. 45, 366–369. 10.1097/MPG.0b013e31806907e8 17873753

[B22] Campagna G. Pesce M. Tatangelo R. Rizzuto A. La Fratta I. Grilli A. (2017). The progression of coeliac disease: its neurological and psychiatric implications. Nutr. Res. Rev. 30, 25–35. 10.1017/S0954422416000214 27976606

[B23] Canales P. Mery V. P. Larrondo F. J. Bravo F. L. Godoy J. (2006). Epilepsy and celiac disease: favorable outcome with a gluten-free diet in a patient refractory to antiepileptic drugs. Neurologist 12, 318–321. 10.1097/01.nrl.0000250950.35887.6c 17122729

[B24] Cantone M. Bramanti A. Lanza G. Pennisi M. Bramanti P. Pennisi G.. (2017). Cortical plasticity in depression. ASN Neuro 9:1759091417711512. 10.1177/1759091417711512 28629225 PMC5480639

[B25] Cantone M. Di Pino G. Capone F. Piombo M. Chiarello D. Cheeran B.. (2014). The contribution of transcranial magnetic stimulation in the diagnosis and in the management of dementia. Clin. Neurophysiol. 125, 1509–1532. 10.1016/j.clinph.2014.04.010 24840904

[B26] Carta M. G. Conti A. Lecca F. Sancassiani F. Cossu G. Carruxi R.. (2015). The burden of depressive and bipolar disorders in Celiac disease. Clin. Pract. Epidemiol. Ment. Health 11, 180–185. 10.2174/1745017901511010180 26962323 PMC4763959

[B27] Carta M. G. Hardoy M. C. Boi M. F. Mariotti S. Carpiniello B. Usai P. (2002). Association between panic disorder, major depressive disorder and celiac disease: a possible role of thyroid autoimmunity. J. Psychosom. Res. 53, 789–793. 10.1016/S0022-3999(02)00328-8 12217453

[B28] Carta M. G. Hardoy M. C. Usai P. Carpiniello B. Angst J. (2003). Recurrent brief depression in celiac disease. J. Psychosom. Res. 55, 573–574. 10.1016/S0022-3999(03)00547-6 14642990

[B29] Casciato S. Morano A. Albini M. Fanella M. Lapenta L. Fattouch J.. (2015). Cryptogenic focal epilepsy and “hidden” celiac disease in adulthood: a causal or accidental link? Int. J. Neurosci. 125, 913–917. 10.3109/00207454.2014.983227 25387071

[B30] Casella S. Zanini B. Lanzarotto F. Ricci C. Marengoni A. Romanelli G.. (2012). Cognitive performance is impaired in coeliac patients on gluten free diet: a case-control study in patients older than 65 years of age. Dig. Liver Dis. 44, 729–735. 10.1016/j.dld.2012.03.008 22484003

[B31] Castillo N. E. Theethira T. G. Leffler D. A. (2015). The present and the future in the diagnosis and management of celiac disease. Gastroenterol Rep (Oxf) 3, 3–11. 10.1093/gastro/gou065 25326000 PMC4324867

[B32] Chin R. L. Latov N. (2005). Peripheral neuropathy and Celiac disease. Curr. Treat. Options Neurol. 7, 43–48. 10.1007/s11940-005-0005-3 15610706

[B33] Chin R. L. Sander H. W. Brannagan T. H. Green P. H. Hays A. P. Alaedini A.. (2003). Celiac neuropathy. Neurology 60, 1581–1585. 10.1212/01.WNL.0000063307.84039.C7 12771245

[B34] Cicarelli G. Della Rocca G. Amboni M. Ciacci C. Mazzacca G. Filla A.. (2003). Clinical and neurological abnormalities in adult celiac disease. Neurol. Sci. 24, 311–317. 10.1007/s10072-003-0181-4 14716525

[B35] Collin P. Pirttilä T. Nurmikko T. Somer H. Erilä T. Keyriläinen O. (1991). Celiac disease, brain atrophy, and dementia. Neurology 41, 372–375. 10.1212/WNL.41.3.372 2006004

[B36] Concerto C. Lanza G. Cantone M. Ferri R. Pennisi G. Bella R.. (2015). Repetitive transcranial magnetic stimulation in patients with drug-resistant major depression: a six-month clinical follow-up study. Int. J. Psychiatry Clin. Pract. 19, 252–258. 10.3109/13651501.2015.1084329 26398527

[B37] Concerto C. Lanza G. Cantone M. Pennisi M. Giordano D. Spampinato C.. (2013). Different patterns of cortical excitability in major depression and vascular depression: a transcranial magnetic stimulation study. BMC Psychiatry 13:300. 10.1186/1471-244X-13-300 24206945 PMC4226249

[B38] Cooke W. T. Smith W. T. (1966). Neurological disorders associated with adult coeliac disease. Brain 89, 683–722. 10.1093/brain/89.4.683 4163580

[B39] Dai A. I. Akcali A. Varan C. Demiryürek A. T. (2014). Prevalence of resistant occipital lobe epilepsy associated with celiac disease in children. Childs. Nerv. Syst. 30, 1091–1098. 10.1007/s00381-014-2387-6 24566676

[B40] Deconinck N. Scaillon M. Segers V. Groswasser J. J. Dan B. (2006). Opsoclonus-myoclonus associated with celiac disease. Pediatr. Neurol. 34, 312–314. 10.1016/j.pediatrneurol.2005.08.034 16638509

[B41] De Santis A. Addolorato G. Romito A. Caputo S. Giordano A. Gambassi G.. (1997). Schizophrenic symptoms and SPECT abnormalities in a coeliac patient: regression after a gluten-free diet. J. Intern. Med. 242, 421–423. 10.1046/j.1365-2796.1997.00200.x 9408073

[B42] Di Lazzaro V. Pilato F. Batocchi A. P. Restuccia D. Cammarota G. Profice P. (2010). Tired legs–a gut diagnosis. Lancet 376, 1798. 10.1016/S0140-6736(10)61163-4 21093652

[B43] Fung V. S. Duggins A. Morris J. G. Lorentz I. T. (2000). Progressive myoclonic ataxia associated with celiac disease presenting as unilateral cortical tremor and dystonia. Mov. Disord. 15, 732–734. 10.1002/1531-8257(200007)15:4<732::AID-MDS1021>3.0.CO;2-J 10928587

[B44] Freeman H. J. (2008). Neurological disorders in adult celiac disease. Can. J. Gastroenterol. 22, 909–911. 10.1155/2008/824631 19018335 PMC2661192

[B45] Gadoth A. Nefussy B. Bleiberg M. Klein T. Artman I. Drory V. E. (2015). Transglutaminase 6 antibodies in the serum of patients with amyotrophic lateral sclerosis. JAMA Neurol. 72, 676–681. 10.1001/jamaneurol.2015.48 25867286

[B46] Gobbi G. Bouquet F. Greco L. Lambertini A. Tassinari C. A. Ventura A.. (1992). Coeliac disease, epilepsy, and cerebral calcifications. the Italian working group on coeliac disease and epilepsy. Lancet 340, 439–443. 10.1016/0140-6736(92)91766-2 1354781

[B47] Green P. H. Alaedini A. Sander H. W. Brannagan T. H. III. Latov N. Chin R. L. (2005). Mechanisms underlying celiac disease and its neurologic manifestations. Cell. Mol. Life Sci. 62, 791–799. 10.1007/s00018-004-4109-9 15868404 PMC11924539

[B48] Green P. H. Jabri B. (2003). Coeliac disease. Lancet 362, 383–391. 10.1016/S0140-6736(03)14027-5 12907013

[B49] Hadjivassiliou M. Aeschlimann P. Strigun A. Sanders D. S. Woodroofe N. Aeschlimann D. (2008). Autoantibodies in gluten ataxia recognize a novel neuronal transglutaminase. Ann. Neurol. 64, 332–343. 10.1002/ana.21450 18825674

[B50] Hadjivassiliou M. Grünewald R. A. Davies-Jones G. A. (2002a). Gluten sensitivity as a neurological illness. J. Neurol. Neurosurg. Psychiatr. 72, 560–563. 10.1136/jnnp.72.5.560 11971034 PMC1737870

[B51] Hadjivassiliou M. Boscolo S. Davies-Jones G. A. Grünewald R. A. Not T. Sanders D. S.. (2002b). The humoral response in the pathogenesis of gluten ataxia. Neurology 58, 1221–1226. 10.1212/WNL.58.8.1221 11971090

[B52] Hadjivassiliou M. Duker A. P. Sanders D. S. (2014). Gluten-related neurologic dysfunction. Handb. Clin. Neurol. 120, 607–619. 10.1016/B978-0-7020-4087-0.00041-3 24365341

[B53] Hadjivassiliou M. Grünewald R. A. Chattopadhyay A. K. Davies-Jones G. A. Gibson A. Jarratt J. A.. (1998). Clinical, radiological, neurophysiological, and neuropathological characteristics of gluten ataxia. Lancet 352, 1582–1585. 10.1016/S0140-6736(98)05342-2 9843103

[B54] Hadjivassiliou M. Grünewald R. A. Lawden M. Davies-Jones G. A. Powell T. Smith C. M. (2001). Headache and CNS white matter abnormalities associated with gluten sensitivity. Neurology 56, 385–388. 10.1212/WNL.56.3.385 11171906

[B55] Hadjivassiliou M. Mäki M. Sanders D. S. Williamson C. A. Grünewald R. A. Woodroofe N. M.. (2006). Autoantibody targeting of brain and intestinal transglutaminase in gluten ataxia. Neurology 66, 373–377. 10.1212/01.wnl.0000196480.55601.3a 16476935

[B56] Hadjivassiliou M. Sanders D. D. Aeschlimann D. P. (2015). Gluten-related disorders: gluten ataxia. Dig. Dis. 33, 264–268. 10.1159/000369509 25925933

[B57] Hadjivassiliou M. Sanders D. S. Grünewald R. A. Woodroofe N. Boscolo S. Aeschlimann D. (2010). Gluten sensitivity: from gut to brain. Lancet Neurol. 9, 318–330. 10.1016/S1474-4422(09)70290-X 20170845

[B58] Hallert C. Aström J. Sedvall G. (1982). Psychic disturbances in adult coeliac disease. III. Reduced central monoamine metabolism and signs of depression. Scand J. Gastroenterol. 17, 25–28. 10.3109/00365528209181039 6182605

[B59] Hanagasi H. A. Gürol E. Sahin H. A. Emre M. (2001). Atypical neurological involvement associated with celiac disease. Eur. J. Neurol. 8, 67–69. 10.1046/j.1468-1331.2001.00155.x 11509083

[B60] Hernanz A. Polanco I. (1991). Plasma precursor amino acids of central nervous system monoamines in children with coeliac disease. Gut 32, 1478–1481. 10.1136/gut.32.12.1478 1773952 PMC1379246

[B61] Holmes G. K. (1996). Non-malignant complications of coeliac disease. Acta Paediatr. Suppl. 412, 68–75. 10.1111/j.1651-2227.1996.tb14257.x 8783765

[B62] Holmes G. K. (2002). Coeliac disease and malignancy. Dig. Liver Dis. 34, 229–237. 10.1016/S1590-8658(02)80198-0 11990397

[B63] Hu W. T. Murray J. A. Greenaway M. C. Parisi J. E. Josephs K. A. (2006). Cognitive impairment and celiac disease. Arch. Neurol. 63, 1440–1446. 10.1001/archneur.63.10.1440 17030661

[B64] Işıkay S. Kocamaz H. Sezer S. Özkars M. Y. Işıkay N. Filik B.. (2015a). The frequency of epileptiform discharges in Celiac disease. Pediatr. Neurol. 53, 78–82. 10.1016/j.pediatrneurol.2015.02.006 26092417

[B65] Işıkay S. Hizli Ş. Çoşkun S. Yilmaz K. (2015b). Increased tissue transglutaminase levels are associated with increased epileptiform activity in electroencephalography among patients with celiac disease. Arq. Gastroenterol. 52, 272–277. 10.1590/S0004-28032015000400005 26840467

[B66] Javed S. Safdar A. Forster A. Selvan A. Chadwick D. Nicholson A.. (2012). Refractory coeliac disease associated with late onset epilepsy, ataxia, tremor and progressive myoclonus with giant cortical evoked potentials–a case report and review of literature. Seizure 21, 482–485. 10.1016/j.seizure.2012.04.003 22565067

[B67] Kaplan J. G. Pack D. Horoupian D. DeSouza T. Brin M. Schaumburg H. (1988). Distal axonopathy associated with chronic gluten enteropathy: a treatable disorder. Neurology 38, 642–645. 10.1212/WNL.38.4.642 2832786

[B68] Karwautz A. Wagner G. Berger G. Sinnreich U. Grylli V. Huber W. D. (2008). Eating pathology in adolescents with celiac disease. Psychosomatics 49, 399–406. 10.1176/appi.psy.49.5.399 18794508

[B69] Labate A. Gambardella A. Messina D. Tammaro S. Le Piane E. Pirritano D.. (2001). Silent celiac disease in patients with childhood localization-related epilepsies. Epilepsia 42, 1153–1155. 10.1046/j.1528-1157.2001.45700.x 11580763

[B70] Lanza G. Bachmann C. G. Ghorayeb I. Wang Y. Ferri R. Paulus W. (2017a). Central and peripheral nervous system excitability in restless legs syndrome. Sleep Med. 31, 49–60. 10.1016/j.sleep.2016.05.010 27745789

[B71] Lanza G. Bella R. Giuffrida S. Cantone M. Pennisi G. Spampinato C.. (2013). Preserved transcallosal inhibition to transcranial magnetic stimulation in nondemented elderly patients with leukoaraiosis. Biomed Res. Int. 2013:351680. 10.1155/2013/351680 23984349 PMC3741902

[B72] Lanza G. Bramanti P. Cantone M. Pennisi M. Pennisi G. Bella R. (2017b). Vascular cognitive impairment through the looking glass of transcranial magnetic stimulation. Behav. Neurol. 2017:1421326. 10.1155/2017/1421326 28348458 PMC5350538

[B73] Lanza G. Cantone M. Lanuzza B. Pennisi M. Bella R. Pennisi G.. (2015a). Distinctive patterns of cortical excitability to transcranial magnetic stimulation in obstructive sleep apnea syndrome, restless legs syndrome, insomnia, and sleep deprivation. Sleep Med. Rev. 19, 39–50. 10.1016/j.smrv.2014.04.001 24849846

[B74] Lanza G. Lanuzza B. Aricò D. Cantone M. Cosentino F. I. Pennisi M.. (2015b). Direct comparison of cortical excitability to transcranial magnetic stimulation in obstructive sleep apnea syndrome and restless legs syndrome. Sleep Med. 16, 138–142. 10.1016/j.sleep.2014.08.016 25534710

[B75] Lebwohl B. Sanders D. S. Green P. H. R. (2017). Coeliac disease. Lancet [Epub ahead of print]. 10.1016/S0140-6736(17)31796-8 28760445

[B76] Licchetta L. Bisulli F. Di Vito L. La Morgia C. Naldi I. Volta U.. (2011). Epilepsy in coeliac disease: not just a matter of calcifications. Neurol. Sci. 32, 1069–1074. 10.1007/s10072-011-0629-x 21630037

[B77] Lichtwark I. T. Newnham E. D. Shepherd S. J. Hosking P. Gibson P. R.. (2014). Cognitive impairment in coeliac disease improves on a gluten-free diet and correlates with histological and serological indices of disease severity. Aliment. Pharmacol. Ther. 40, 160–170. 10.1111/apt.12809 24889390

[B78] Lionetti E. Francavilla R. Pavone P. Pavone L. Francavilla T. Pulvirenti A.. (2010). The neurology of coeliac disease in childhood: what is the evidence? A systematic review and meta-analysis. Dev. Med. Child Neurol. 52, 700–707. 10.1111/j.1469-8749.2010.03647.x 20345955

[B79] Lu C. S. Thompson P. D. Quinn N. P. Parkes J. D. Marsden C. D. (1986). Ramsay Hunt syndrome and coeliac disease: a new association? Mov. Disord. 1, 209–219. 10.1002/mds.870010306 3504245

[B80] Ludvigsson J. F. Mariosa D. Lebwohl B. Fang F. (2014). No association between biopsy-verified celiac disease and subsequent amyotrophic lateral sclerosis–a population-based cohort study. Eur. J. Neurol. 21, 976–982. 10.1111/ene.12419 24708265 PMC4057356

[B81] Ludvigsson J. F. Zingone F. Tomson T. Ekbom A. Ciacci C. (2012). Increased risk of epilepsy in biopsy-verified celiac disease: a population-based cohort study. Neurology 78, 1401–1407. 10.1212/WNL.0b013e3182544728 22517096

[B82] Luostarinen L. Himanen S. L. Luostarinen M. Collin P. Pirttilä T. (2003). Neuromuscular and sensory disturbances in patients with well treated coeliac disease. J. Neurol. Neurosurg. Psychiatr. 74, 490–494. 10.1136/jnnp.74.4.490 12640070 PMC1738407

[B83] Lurie Y. Landau D. A. Pfeffer J. Oren R. (2008). Celiac disease diagnosed in the elderly. J. Clin. Gastroenterol. 42, 59–61. 10.1097/01.mcg.0000247995.12087.7b 18097291

[B84] Magaudda A. Dalla Bernardina B. De Marco P. Sfaello Z. Longo M. Colamaria V.. (1993). Bilateral occipital calcification, epilepsy and coeliac disease: clinical and neuroimaging features of a new syndrome. J. Neurol. Neurosurg. Psychiatr. 56, 885–889. 10.1136/jnnp.56.8.885 8350105 PMC1015143

[B85] McKeon A. Lennon V. A. Pittock S. J. Kryzer T. J. Murray J. (2014). The neurologic significance of celiac disease biomarkers. Neurology 83, 1789–1796. 10.1212/WNL.0000000000000970 25261501 PMC4240435

[B86] Morello F. Ronzani G. Cappellari F. (2003). Migraine, cortical blindness, multiple cerebral infarctions and hypocoagulopathy in celiac disease. Neurol. Sci. 24, 85–89. 12827546 10.1007/s100720300079

[B87] Mumford C. J. Fletcher N. A. Ironside J. W. Warlow C. P. (1996). Progressive ataxia, focal seizures, and malabsorption syndrome in a 41 year old woman. J. Neurol. Neurosurg. Psychiatr. 60, 225–230. 10.1136/jnnp.60.2.225 8708663 PMC1073814

[B88] Niederhofer H. Pittschieler K. (2006). A preliminary investigation of ADHD symptoms in persons with celiac disease. J. Atten. Disord. 10, 200–204. 10.1177/1087054706292109 17085630

[B89] Nikpour S. (2012). Neurological manifestations, diagnosis, and treatment of celiac disease: a comprehensive review. Iran J. Neurol. 11, 59–64. 24250863 PMC3829244

[B90] Parisi P. Pietropaoli N. Ferretti A. Nenna R. Mastrogiorgio G. Del Pozzo M.. (2015). Role of the gluten-free diet on neurological-EEG findings and sleep disordered breathing in children with celiac disease. Seizure 25, 181–183. 10.1016/j.seizure.2014.09.016 25457448

[B91] Parisi P. Principessa L. Ferretti A. D'Onofrio D. Del Giudice E. Pacchiarotti C.. (2014). “EEG abnormalities” may represent a confounding factor in celiac disease: a 4-year follow-up family report. Epilepsy Behav. Case Rep. 2, 40–42. 10.1016/j.ebcr.2014.01.008 25667866 PMC4307964

[B92] Paulus W. Classen J. Cohen L. G. Large C. H. Di Lazzaro V. Nitsche M.. (2008). State of the art: pharmacologic effects on cortical excitability measures tested by transcranial magnetic stimulation. Brain Stimul. 1, 151–163. 10.1016/j.brs.2008.06.002 20633382

[B93] Pawlak-Osińska K. Kaźmierczak H. Kuczyńska R. Szaflarska-Popławska A. (2007). Looking for the auditory and vestibular pathology in celiac disease. Otolaryngol. Pol. 61, 178–183. 10.1016/S0030-6657(07)70409-2 17668806

[B94] Pellecchia M. T. Scala R. Perretti A. De Michele G. Santoro L. Filla A.. (1999). Cerebellar ataxia associated with subclinical celiac disease responding to gluten-free diet. Neurology 53, 1606–1608. 10.1212/WNL.53.7.1606-a 10534283

[B95] Pennisi G. Bella R. Lanza G. (2015). Motor cortex plasticity in subcortical ischemic vascular dementia: what can TMS say? Clin. Neurophysiol. 126, 851–852. 10.1016/j.clinph.2014.09.001 25270240

[B96] Pennisi G. Ferri R. Cantone M. Lanza G. Pennisi M. Vinciguerra L.. (2011a). A review of transcranial magnetic stimulation in vascular dementia. Dement. Geriatr. Cogn. Disord. 31, 71–80. 10.1159/000322798 21242688

[B97] Pennisi G. Ferri R. Lanza G. Cantone M. Pennisi M. Puglisi V.. (2011b). Transcranial magnetic stimulation in Alzheimer's disease: a neurophysiological marker of cortical hyperexcitability. J. Neural. Transm. (Vienna) 118, 587–598. 10.1007/s00702-010-0554-9 21207079

[B98] Pennisi G. Lanza G. Giuffrida S. Vinciguerra L. Puglisi V. Cantone M.. (2014). Excitability of the motor cortex in *de novo* patients with celiac disease. PLoS ONE 9:e102790. 10.1371/journal.pone.0102790 25062250 PMC4111288

[B99] Pennisi M. Lanza G. Cantone M. Ricceri R. Ferri R. D'Agate C. C.. (2017). Cortical involvement in celiac disease before and after long-term gluten-free diet: a transcranial magnetic stimulation study. PLoS ONE 12:e0177560. 10.1371/journal.pone.0177560 28489931 PMC5425211

[B100] Pennisi M. Lanza G. Cantone M. Ricceri R. Spampinato C. Pennisi G.. (2016). Correlation between motor cortex excitability changes and cognitive impairment in vascular depression: pathophysiological insights from a longitudinal TMS study. Neural Plast. 2016:8154969. 10.1155/2016/8154969 27525127 PMC4971324

[B101] Pratesi R. Modelli I. C. Martins R. C. Almeida P. L. Gandolfi L. (2003). Celiac disease and epilepsy: favorable outcome in a child with difficult to control seizures. Acta Neurol. Scand. 108, 290–293. 10.1034/j.1600-0404.2003.00082.x 12956865

[B102] Ranua J. Luoma K. Auvinen A. Mäki M. Haapala A. M. Peltola J.. (2005). Celiac disease-related antibodies in an epilepsy cohort and matched reference population. Epilepsy Behav. 6, 388–392. 10.1016/j.yebeh.2005.01.007 15820348

[B103] Sabel'nikova E. A. Krums L. M. Parfenov A. I. Vorob'eva N. N. Gudkova R. B. (2013). Specific features of rehabilitation in patients with gluten-sensitivity celiac disease. Ter. Arkh. 85, 42–47. 23536945

[B104] Sallem F. S. Castro L. M. Jorge C. Marchiori P. Barbosa E. (2009). Gluten sensitivity presenting as myoclonic epilepsy with cerebellar syndrome. Mov. Disord. 24, 2162–2163. 10.1002/mds.22576 19705357

[B105] Sarrigiannis P. G. Hoggard N. Aeschlimann D. Sanders D. S. Grünewald R. A. Unwin Z. C.. (2014). Myoclonus ataxia and refractory coeliac disease. Cerebellum Ataxia 1:11. 10.1186/2053-8871-1-11 26331035 PMC4552176

[B106] Schuppan D. Junker Y. Barisani D. (2009). Celiac disease: from pathogenesis to novel therapies. Gastroenterology 137, 1912–1933. 10.1053/j.gastro.2009.09.008 19766641

[B107] Siqueira Neto J. I. Costa A. C. Magalhães F. G. Silva G. S. (2004). Neurological manifestations of celiac disease. Arq. Neuropsiquiatr. 62, 969–972. 10.1590/S0004-282X2004000600007 15608953

[B108] Spampinato C. Aguglia E. Concerto C. Pennisi M. Lanza G. Bella R.. (2013). Transcranial magnetic stimulation in the assessment of motor cortex excitability and treatment of drug-resistant major depression. IEEE Trans. Neural Syst. Rehabil. Eng. 21, 391–403. 10.1109/TNSRE.2013.2256432 23559064

[B109] Tijssen M. A. Thom M. Ellison D. W. Wilkins P. Barnes D. Thompson P. D.. (2000). Cortical myoclonus and cerebellar pathology. Neurology 54, 1350–1356. 10.1212/WNL.54.6.1350 10746609

[B110] Tison F. Arne P. Henry P. (1989). Myoclonus and adult coeliac disease. J. Neurol. 236, 307–308. 10.1007/BF00314464 2760650

[B111] Turner M. R. Chohan G. Quaghebeur G. Greenhall R. C. Hadjivassiliou M. Talbot K. (2007). A case of celiac disease mimicking amyotrophic lateral sclerosis. Nat. Clin. Pract. Neurol. 3, 581–584. 10.1038/ncpneuro0631 17914346

[B112] Turner M. R. Goldacre R. Ramagopalan S. Talbot K. Goldacre M. J. (2013). Autoimmune disease preceding amyotrophic lateral sclerosis: an epidemiologic study. Neurology 81, 1222–1225. 10.1212/WNL.0b013e3182a6cc13 23946298 PMC3795611

[B113] Tursi A. Giorgetti G. M. Iani C. Arciprete F. Brandimarte G. Capria A.. (2006). Peripheral neurological disturbances, autonomic dysfunction, and antineuronal antibodies in adult celiac disease before and after a gluten-free diet. Dig. Dis. Sci. 51, 1869–1874. 10.1007/s10620-005-9054-4 16967315

[B114] Usai P. Serra A. Marini B. Mariotti S. Satta L. Boi M. F.. (2004). Frontal cortical perfusion abnormalities related to gluten intake and associated autoimmune disease in adult coeliac disease: 99mTc-ECD brain SPECT study. Dig. Liver Dis. 36, 513–518. 10.1016/j.dld.2004.03.010 15334770

[B115] Usanova E. P. Shapkina O. A. Matkivskiǐ R. A. Fedulova É. N. Uspenskaia I. D. (2012). The organization of comprehensive rehabilitation of the children presenting with inflammatory intestinal diseases and celiacia under the conditions of a health resort. Vopr. Kurortol. Fizioter. Lech. Fiz. Kult. 4, 37–40. 22994063

[B116] Uygur-Bayramicli O. Ozel A. M. (2011). Celiac disease is associated with neurological syndromes. Dig. Dis. Sci. 56, 1587–1588. 10.1007/s10620-011-1663-5 21409373

[B117] van Hees N. J. Van der Does W. Giltay E. J. (2013). Coeliac disease, diet adherence and depressive symptoms. J. Psychosom. Res. 74, 155–160. 10.1016/j.jpsychores.2012.11.007 23332531

[B118] Vieira C. Jatobá I. Matos M. Diniz-Santos D. Silva L. R. (2013). Prevalence of celiac disease in children with epilepsy. Arq. Gastroenterol. 50, 290–296. 10.1590/S0004-28032013000400010 24474232

[B119] Yelland G. W. (2017). Gluten-induced cognitive impairment (“brain fog”) in coeliac disease. J. Gastroenterol. Hepatol. 32, 90–93. 10.1111/jgh.13706 28244662

[B120] Zelnik N. Pacht A. Obeid R. Lerner A. (2004). Range of neurologic disorders in patients with celiac disease. Pediatrics 113, 1672–1676. 10.1542/peds.113.6.1672 15173490

[B121] Ziemann U. Reis J. Schwenkreis P. Rosanova M. Strafella A. Badawy R.. (2015). TMS and drugs revisited 2014. Clin. Neurophysiol. 126, 1847–1868. 10.1016/j.clinph.2014.08.028 25534482

[B122] Zingone F. Siniscalchi M. Capone P. Tortora R. Andreozzi P. Capone E.. (2010). The quality of sleep in patients with coeliac disease. Aliment. Pharmacol. Ther. 32, 1031–1036. 10.1111/j.1365-2036.2010.04432.x 20937049

